# Clinical and epidemiological features of imported loiasis in Beijing: a report from patients returned from Africa

**DOI:** 10.1186/s12879-024-09620-6

**Published:** 2024-07-20

**Authors:** Xiaoli Li, Minjun Huang, Kuo Bi, Yang Zou, Fei Wang, Xiaoyan Zheng, Lei Wang

**Affiliations:** 1 Beijing Institute of Tropical Medicine, Beijing Friendship Hospital, Capital Medical University, Beijing, 100050 China; 2 Beijing Key Laboratory for Research on Prevention and Treatment of Tropical Diseases, Beijing, 100050 China; 3 Department of Pathology, Beijing Friendship Hospital, Capital Medical University, Beijing, 100050 China

**Keywords:** Loiasis, *Loa loa*, Filariasis, Eosinophilia, Travel, Imported

## Abstract

**Background:**

Loiasis is one of the significant filarial diseases for people living in West and Central Africa with wide endemic area but is not seen in China. As economy booms and international traveling increase, China faces more and more imported parasitic diseases that are not endemic locally. Loiasis is one of the parasitic diseases that enter China by travelers infected in Africa. The better understanding of the clinical and laboratory features of *loa loa* infection will facilitate the diagnosis and treatment of loiasis in China.

**Methods:**

The study targeted travelers who were infected with *L. loa* in endemic Africa regions and returned to Beijing between 2014 and 2023. Epidemiological, clinical, and biological data as well as treatment of these patients were collected.

**Results:**

Total 21 cases were identified as *L. loa* infection based on their typical clinical manifestations and parasite finding. All cases had a history of travel to Africa for more than 6 months, most of them are the construction workers dispatched to West Africa with outdoor activities. Calabar swelling (*n* = 19; 90.5%) and pruritus (*n* = 11; 52.4%) were among the most common clinical symptoms followed by muscle pain (*n* = 7; 33.3%) and skin rash (*n* = 2; 9.5%). The adult worms were observed in the eyelid or subconjunctiva (*n* = 2; 9.5%) and subcutaneous tissues (*n* = 2; 9.5%). Although all patients presented with a high eosinophil count (> 0.52 × 10^9^/L), only two cases displayed microfilariae in fresh venous blood and positive for filarial antigen. A cut section of adult worm was observed through biopsy on a skin nodule surrounded by lymphocytes, plasma cells and eosinophils. All subjects were positive in PCR targeting *L. loa* ITS-1. The constructed phylogenetic tree based on the amplified ITS-1 sequences identified their genetical relation to the *L. Loa* from Africa. All patients treated with albendazole and diethylcarbamazine were recovered without relapse.

**Conclusion:**

This study provides useful information and guideline for physicians and researchers in non-endemic countries to diagnose and treat loiasis and *L. loa* infections acquired from endemic regions.

**Supplementary Information:**

The online version contains supplementary material available at 10.1186/s12879-024-09620-6.

## Introduction

*Loa loa*, also called African eye worm, is a filarial nematode that causes loiasis in the tropical areas of West and Central Africa including Cameroon, Congo, Gabon and Nigeria [1]. It is estimated that more than 14 million people live in high-risk areas, at least 10 million people are infected with *Loa loa* filariae and over 6 million cases require treatment by 2025 [2]. This parasitic disease is transmitted via the repeated bites of deerflies (mainly *Chrysops silacea* and *C. dimidiata*) [3]. Pathognomonic symptom of loiasis is Calabar swelling which is marked by painful and itchy, migratory, localized angio-oedema around the joints or in the face. The adult worm migration can be visible in the eye or under the thin skin [4]. The complications affecting different organ compartments such as the central nervous system, kidneys, heart and lungs have been reported in some cases [5].

China used to be a country with serious endemic of lymphatic filariasis caused by filarial nematodes *Wuchereria bancrofti* and *Brugia malayi* transmitted by the mosquito bite [6]. Over the decade’s efforts with mass treatment and vector control, lymphatic filariasis has been fully controlled and the elimination of this disease nationwide has been announced in 2007 [7]. Loiasis is not endemic in China and the *L. loa* filaria has never be detected in native residents or vectors, but occasionally detected in the international travelers (e.g., tourists, dispatched workers and migrants) from West Africa [8]. As economy quickly grows in China and globalization advances, more and more people are traveling in and out of China, including tourists, businessmen, immigrants and migrant workers. Statistically, 122 million Chinese people travelled abroad in 2016 [9] including more than 1 million migrant workers working in Africa [10]. Since most of Chinese have not ever been exposed to *L. loa* filaria, they are highly susceptible to the infection of this nematode when they move or travel to the endemic areas. Loiasis has been considered as one of the most common imported parasitic diseases followed by malaria and schistosomiasis in China from 2008 to 2016 [11]. During the last few years, the imported loiasis cases have been reported in countries including Belgium [12], London [13], France [14], Spain [15], Italy [16], Japan [17] and China [18]. Limited information concerning the epidemiologic characteristics could be observed in mainland China from the National Notifiable Disease Report System (NNDRS). There have been five reported instances of *L. Loa* infection across China, with the exception of Beijing in the existing literature. These cases were recorded in the Sichuan, Zhejiang, Shanghai, and Guangxi provinces [18–22]. Although China has no native case of loiasis, it is expected that more and more imported loiasis cases will be seen due to the large number of migrant workers who are dispatched to and return from Africa.

Although more imported cases of loiasis have been seen in migrant workers returned from Africa, there is no detail and comprehensive study regarding the clinical features of the imported loiasis reported in China. Herein, to improve the awareness of this rare parasitic disease in China, we collected 21 cases diagnosed with imported loiasis in our hospital in Beijing between 2014 and 2023 and the clinical manifestations, pathologic characterization of *L. loa* infection in these Chinese migrant workers returned from endemic Africa were analyzed.

## Materials and methods

### Study design, diagnostic procedures and inclusion criteria

This retrospective study was conducted on the imported loiasis cases admitted to the Beijing Friendship Hospital, Capital Medical University between July 2014 and July 2023.

The definitive diagnosis of loiasis was made based on the combination of clinical manifestations, traveling history in the endemic areas and the parasite identification [23] as below:

- Clinical manifestations: Patients present typical Calabar swellings (recurrent painful oedema of the extremities), skin pruritus, arthralgia, myalgia and hypereosinophilia (eosinophilic count > 0.52 × 10^9^/L).
- Parasitological identification: Patients with positive microfilaraemia or with documented migration of adult *L. loa* worm(s) in the eyelid, subconjunctiva or under skin (biopsy).
- Molecular methods: PCR positive for *L. loa* DNA in patients’ blood.
- Epidemiological evidence: Patients with history of visiting or living in endemic areas outside China, primarily in Africa.

Parasitological identification was made by the finding of microfilariae in peripheral blood smear or the presence of adult worms in the eyelid, subconjunctiva or under skin by biopsy examination. The load of microfilaraemia was quantified by thick blood film technique using peripheral blood collected around midday (between 10 AM and 2 PM) reflecting the periodicity of the infection [24].

To exclude the possibility of cross-reactivity with other lymphatic filaria, the filarial antigenemia were detected by immunochromatographic card test (BinaxNOW; Alere Scarborough Inc., Scarborough, ME) used for immunological detection of soluble *Wuchereria bancrofti* antigens in peripheral blood [25].

**Fig. 1:**
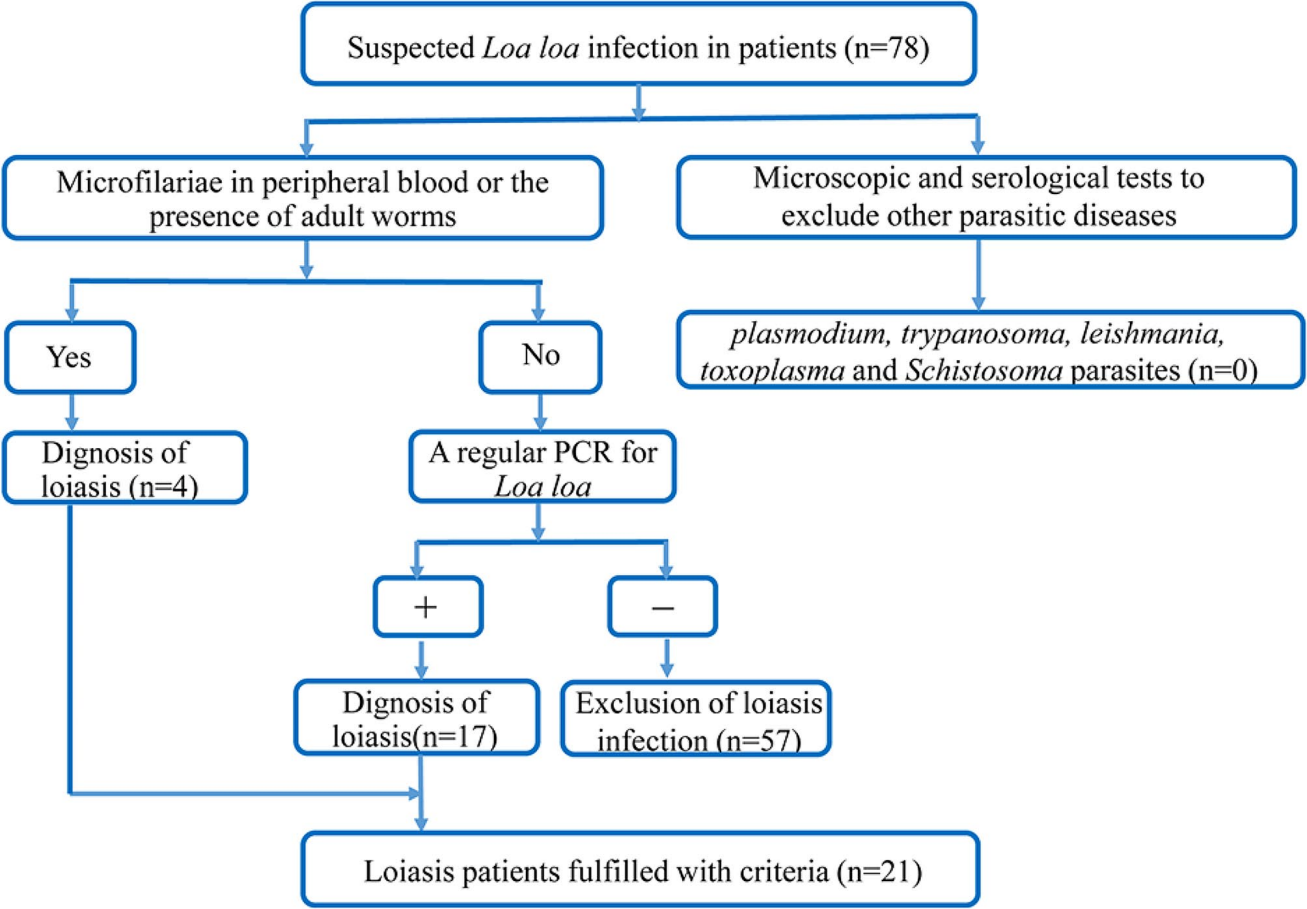
Flow chart for the diagnosis of loiasis

To exclude the possible infection of other parasitic diseases co-endemic in the same areas, the microscopic and serological tests were also performed for the detection of *Plasmodium*, *trypanosoma*, *leishmania* spp., *toxoplasma* and *Schistosoma* parasites. The fecal examination was also performed for all patients and there were no helminthic eggs found (Fig. 1).

### Information collected

The epidemiological information (age, gender, traveled countries), clinical manifestations including major symptoms and complaints and laboratory or parasitical examination results including blood cells count, antibody detection and microfilariae count, were collected from each patient and input into database.

### Histopathology and immunohistochemistry of skin lesion

Biopsy was made on a skin nodule located on the right forearm of a patient returned from Republic of Congo. The skin module tissue was fixed in 4% formalin, embedded in paraffin, cut into tissue sections, and stained with hematoxylin-eosin (H&E). For immunohistochemical staining, the tissue sections were stained with immunohistochemical reagents including anti-CD4, anti-CD8, anti-CD19 and anti-CD56 antibodies (Becton Dickinson, San Jose, California).

### PCR amplification and phylogenetic analysis

A regular polymerase chain reaction (PCR) was used to detect *L. loa* microfilariae DNA in blood [26]. Briefly, DNA was extracted from peripheral blood samples using a DNA extraction kit (TIANGEN, DP705, Beijing, CHN). The primers were designed based on the internal transcribed spacer region 1 (ITS1) sequence of the *L. loa* ribosomal RNA (Table 1) [27]. The amplification of housekeeping gene GAPDH was used as positive control. The cycling conditions for PCR were 95 °C for 9 min, then 30 cycles of 94 °C for 30 s, 58 °C for 30 s, and 72 °C for 45 s followed by 1 cycle of 72 °C for 10 min. The PCR products were analyzed by electrophoresis in 2% agarose gel (Conda, Spain). The purified PCR products were sent to Ruibiotech Company (Beijing, China) for DNA sequencing.

**Table 1:** Primer sequences for PCR

Gene name	Forward (5′-3′)	Reverse (5′-3′)
ITS1	GGTGAACCTGCGGAAGGATC	CTCAATGCGTCTGCAATTCGC
GAPDH	GATTCCACCCATGGCAAATTC	CTGGAAGATGGTGATGGGATT

The phylogenetic tree was constructed based on ITS1 sequences amplified from patients’ peripheral blood and those known *L. loa* ITS1 sequences deposited in the NCBI GenBank. The sequences were aligned by the ClustalW method with MEGA software (version 11) [28] and the phylogenetic analysis was done based on neighbor-joining (NJ) method with bootstrap support (1000 replicates).

### Treatment regimen and follow-up

Given the risk of serious adverse events after diethylcarbamazine (DEC) or ivermectin (IVM) treatment which are the essential drugs to treat filariasis, the diagnosed patients were given a course of albendazole (ABZ, 400 mg thrice daily for 10 days) to reduce the load of *Loa microfilaraemia* before starting with DEC treatment (6 mg/kg/d divided in two to three doses for 21d). Repeated DEC therapy is required for patients with severe manifestations, high eosinophilia, or positive for parasite detections as above. The clinical symptoms and laboratory parameters were followed up regularly in these patients before starting additional treatment cycle.

### Statistical analysis

Statistical analyses were conducted using SPSS version 21.0 (IBM SPSS Statistics 22; Armonk, NY). Continuous variables were described as mean ± standard deviations while categorical variables were expressed as frequencies and percentages.

## Results

### Demographic features

During 2014–2023, 78 cases suspected with *L. loa* infection returned from endemic regions in Africa were admitted in our hospital. Among them, all cases were ruled out for other parasitic infections including *plasmodium*,* trypanosoma*,* leishmania*,* toxoplasma* and *Schistosoma* parasites which are co-endemic in the regions with *L. loa* infection by specific parasite or antibody examination. Subsequently, 57 patients were excluded from analysis due to the lack of positive findings, neither microfilaraemia nor PCR positive in blood. The rest 21 patients with confirmed *L. loa* infection met the inclusion criteria (Fig. 1). Patients’ epidemiological characteristics are outlined in Table 2. Twenty patients (95.2%) were male with age ranged from 24 to 53 (mean age of 38.19 ± 9.58 years). All patients had explicit travel history to West and Central Africa, including Cameroon, Gabon, Republic of Congo, Democratic Republic of the Congo (DRC), Equatorial Guinea and Sudan, where the *L. loa* infection is endemic. Most cases (95.2%) had living history in the endemic regions for more than 6 months during the past years. Most cases were involved with outdoor work or activities such as building or infrastructure construction, farming or drivers, that allow them to have chance to be bitten by deerflies. All cases reported to be bitten by mosquito, flies or other insects on exposed areas of the body during their staying in the regions.

**Table 2:** Sociodemographic characteristics of patients with loiasis during 2014–2023

Items	Loiasis (*N* = 21)	Percent (%)
Gender		
Male	20	95.2
Female	1	4.8
Age (years)		/
Mean (SD)	38.19(9.58)	/
Median (Min, Max)	39 (24,53)	/
Region of Exposure		
Cameroon	6	28.6
Gabon	5	23.8
Republic of Congo	5	23.8
Democratic Republic of the Congo (DRC)	2	9.5
Equatorial Guinea	2	9.5
Sudan	1	4.8
Type of travel		
Africa short-time traveler ≤ 6 M	1	4.8
Africa long-time traveler > 6 M	20	95.2
Occupation		
Labor workers	10	47.6
Enterprise/business/service personnel	4	19.1
Driver	4	19.1
Translator	3	14.3
History of fly or other insect bite	21	100
Death	0	0

### Clinical manifestations

In Table 3, the most common symptom that patients complained (19/21) was the Calabar swelling characterized by the angioedema on the limb extremities, especially on ankles (*N* = 5), wrists (*N* = 7), or arms and legs (*N* = 7). More than half of the cases complained itch on their skin all over the body (pruritus) (52.4%), followed by muscle and joint pain (33.3%), and skin rash on the trunk and extremities (9.5%). Worm crawling under the eyelids or subcutaneous tissue was relatively uncommon. A case reported subconjunctival worm moving in right eye and another case reported in binocular eyelids. These cases with eye worms complained ocular discomfort, a noticeable foreign object sensation without conjunctival haemorrhage and diminution of vision. One patient had subcutaneous nodule as a serpiginous cord in right forearm, and another one had worm migration in subcutaneous tissue of the left anterior chest wall.

**Table 3:** Clinical characteristics of patients with loiasis

Variables	Loiasis (*N* = 21)	Percent (%)
Eosinophilia	21	100
Calabar swelling (ankles, wrists, or arms and legs)	19	90.5
Pruritus	11	52.4
Pain	7	33.3
Skin rash	2	9.5
Adult worm migration		
Eyelids or subconjunctival	2	9.5
Subcutaneous	2	9.5
Asymptomatic	0	0
Microfilaraemia	2	9.5
Delay of diagnosis (months)		
Mean (SD)	13.10 (12.74)	-
Median (Min, Max)	7 (1, 48)	-

The laboratory microfilaraemia positive rate was such low; only 2 of 21 were microfilaremic. Due to the low rate of parasite finding, most of loiasis diagnosis was delayed from 1 to 48 months with mean delay period of 13.10 months.

### Laboratory and parasitological examination

Considering that hypereosinophilia is associated with invasive helminthic infections, every suspected patient returned from the endemic regions received multiple routine blood tests and fecal examination. Significantly, all patients presented eosinophilia in their blood test with an absolute eosinophil count > 0.52 × 10^9^/L, but with normal levels of erythrocyte and platelet (Table 4). The liver enzymes and serum globulin level were within the normal range.

**Table 4:** Laboratory findings of patients with loiasis (the positive results are shown in bold)

Variables	Mean(Max, Min)	Normal range
Routine blood tests		
Leukocytes (×10^9^/L)	11.52(6.53, 25.75)	3.50–9.50
Red blood count(×10^12^/L)	4.78(3.91, 5.13)	4.30–5.80
Hemoglobin (g/L)	147.68(116, 176)	130–175
Eosinophils (×10^9^/L)	**6.51(0.54**,** 40.30)**	**0.02–0.52**
Platelet (×10^9^/L)	211.47(129, 325)	125–350
Liver function tests		
ALT(U/L)	26.79(7, 68)	9–50
AST(U/L)	20.57(9.40, 33.20)	15–40
Albumin(g/L)	41.83(36, 69.20)	40–55
Globulin (g/L)	29.35(20.40, 35.30)	20–40
Etiology		
*Loa loa* microfilariae (mf) densities (mf/mL)	**3.30 × 10**^**5**^**(1.80 × 10**^**5**^, **4.68 × 10**^**5**^**)**	**0**
Filarial antigenemia (%)	**9.5**	**Neg**
ITS1 PCR positive (%)	**100**	**Neg**

**Fig. 2:**
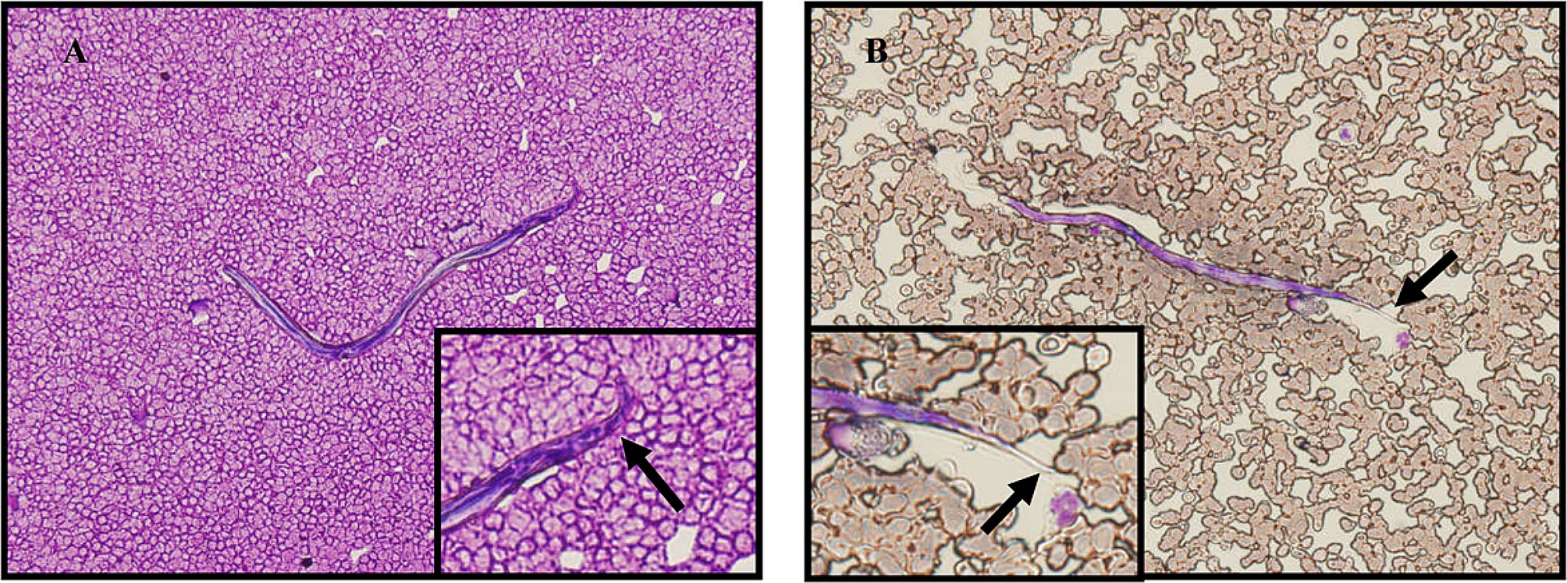
Morphology of microfilariae in blood smear. **(A)** Thin blood film smear stained by Wright-Giemsa showing *L. loa* microfilariae, caudal nuclei extended to the tip of the tail (arrows), **(B)** The translucid sheath of microfilariae in lightly stained film (arrows). 400 × magnification

The definitive diagnosis of *L. loa* infection is based on the presence of microfilariae in blood. However, in this study, only two cases displayed microfilariae in fresh venous blood obtained in daytime (Fig. 2A and B). The filarial antigen test was positive for 2 of 21 patients (9.5%). Significantly, all patients showed positive in PCR test targeting *L. loa* ITS1 with 457 bp products (Additional file 1: Fig. S1 A and B). The amplified PCR products were DNA sequenced.

### Histopathologic features and immunohistochemistry

**Fig. 3:**
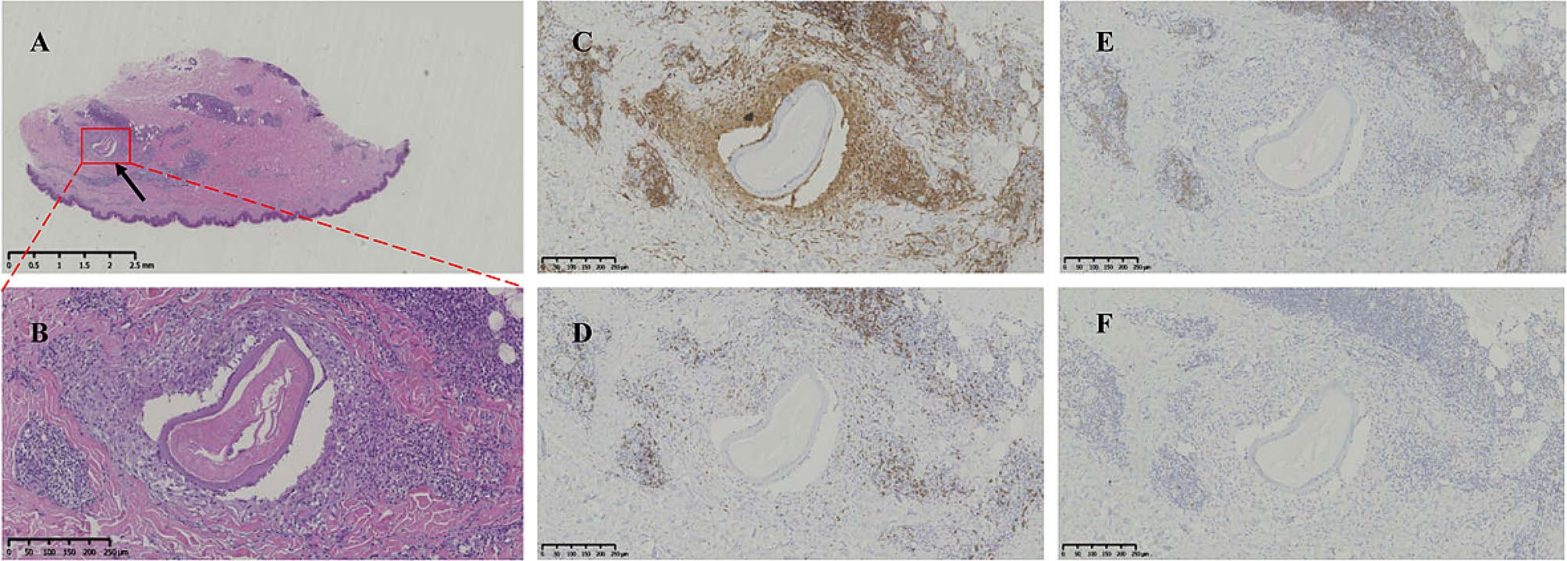
Histopathologic features and immunohistochemistry of skin module infected with *L. loa* adult worm. Hematoxylin-eosin stained section of skin nodule tissue under microscope exhibited a transection of adult worm (**A**, arrows, 10 × magnification) surrounded by the granuloma, lymphocytes, plasma cells and eosinophils (**B**, 100 × magnification). There are many CD4 lymphocyte filtrated around the worm (**C**, 100 × magnification), less CD8 (**D**, 100 × magnification) and few CD 19 (**E**, 100 × magnification) and CD 56 positive cells in the granuloma (**F**, 100 × magnification)

Biopsy was performed on a patient with obvious skin module located on the right forearm. The histological examination on the biopsy section showed a transection of an adult worm surrounded by the granuloma and inflammatory cells infiltrated including many eosinophils (Fig. 3A and B). The immunohistochemistry staining showed the significant filtration of CD4 lymphocytes followed by the CD8 cells in the granuloma surrounding the parasite section. There were few CD19 and CD56 cells filtration (Fig. 3 C-F).

### Phylogenetic analysis

**Fig. 4:**
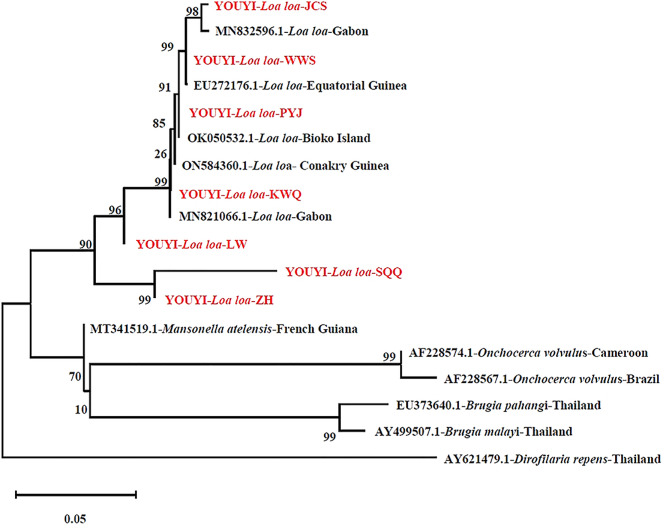
Phylogenetic tree of the ITS-1 sequences of *L. loa* (red font) obtained from 7 Chinese migrant workers returned from endemic Africa. Phylogenetic tree was conducted by MEGA11 software using the neighbor-joining method and 1000 bootstrap values

A phylogenetic tree was constructed based on the seven sequences of IST1 PCR products obtained from seven patient blood samples in this study, compared with known 5 sequences of *L. Loa* ITS1 from NCBI GenBank, represented from Equatorial Guinea and Gabon through the neighbor-joining method, where *Dirofilaria repens* (AY621479.1) acts as an outgroup. These obtained sequences clustered closely with each other and other previously described *L. Loa* sequences, but are distinct from *Dirofilaria repens* sequences, indicating their origin in West and Central Africa (Fig. 4).

### Treatment and outcomes

All patients received albendazole (ABZ) then diethylcarbamazine (DEC) combination therapy once diagnosis of loiasis was confirmed. The dosage of ABZ was 400 mg 3 times/day for 10 days, and then of DEC 6 mg/kg/day for the following 21 days. One or more courses of DEC were given if the symptoms or eosinophilia remain or the PCR keeps positive. All cases were received anti-allergic therapy (loratadine) alone with the anti-parasite chemotherapy to avoid Mazzotti reaction. Neither encephalitis nor any other adverse reactions were observed in any patients. Consequently, 21 patients were fully recovered and no further persistence or relapse of symptoms or eosinophilia were reported (Table 5).

**Table 5:** Posttreatment symptoms following initial treatment of patients with loiasis

Variables	Loiasis (*N* = 21)	Percent (%)
Clinical examination		
Calabar swelling	0	0
Pruritus	0	0
Pain or skin rash	0	0
Adult worm migration	0	0
Adverse reactions	0	0
Laboratory findings		
Eosinophilia	0	0
Filarial antigenemia		
Positive	0	0
Negative	21	100
Microfilaraemia		
Positive	0	0
Negative	21	100
Conventional PCR positive		
Positive	0	0
Negative	21	100

## Discussion

Loiasis is one of vector-borne diseases which have brought a serious threat to global public health [29]. It threats not only local people living in the endemic areas but the travelers who are infected through the bite by the flies carrying the infective larvae [1]. More than 30 million are exposed and at risk of infection worldwide [1]. The global warming and increased international travelers to the endemic regions increase the infection opportunity and the spread of *L. loa* infections. As a vector-borne disease, loiasis has been recognized as an imported infection among visitors and migrants returned from endemic regions. Based on the report from international GeoSentinel Surveillance Network, the most acquired infections for travelers and immigrants/refugees from endemic regions were filaria, 25% of them were infections of *L. loa* [30]. Since free-market reforms and opening up to foreign trade and investment in 1979, China has been among the world’s fastest-growing economies. As increased international economic activities and travelling, China faces more and more threat of imported parasitic infections, especially malaria, filariasis and leishmaniasis. In this study, we described 21 cases of *L. lo*a infection imported from West Africa enrolled in our department during the past 10 years. All of them were Chinese people with a travel history to sub-Saharan Africa for longer than 6 months. While longer stay in endemic area appears more likely to acquire filarial infections, it was found that infection acquisition was high for travelers who had travel durations between 1 and 6 months [30]. It is consistent with our result that 20 out of 21 patients stayed in the endemic countries for more than 6 months. In addition to the travel region and the duration of trip, outdoor exposure is a major factor related to the risk of filarial infection, which increases the chance of insect bites which transmits infections of vector-borne viruses, bacteria, protozoa, or nematodes [31]. In this study, most of cases are engineering technicians and construction workers engaged in the outdoor infrastructure construction in the endemic countries who complained to be frequently bitten by the blood sucking flies. The prolonged exposure to the insect bites outdoor increases the chance to acquire vectored borne parasitic infections [32].

Clinical manifestations of filarial infections are usually subtle, since symptoms are often benign and transient. Infected local residents may remain asymptomatic for days or even years, but for those travelers who have no immunity, loiasis is most often symptomatic [14]. Calabar swellings is a transient subcutaneous swelling tracking the migratory course of the adult *L. loa* filaria through the tissues caused by the hypersensitivity response in subcutaneous tissues against parasite antigens. The appearance rate of Calabar swellings in local people in Southeast Gabon was 17.98%, however, these swellings were observed in 63% cases of imported loiasis in France [33]. In this study, 19 out of 21 cases appeared with Calabar swellings happened in both upper and lower limbs especially in ankles and wrists, further confirming that travelers have higher rate of Calabar swellings than local people in endemic regions as the common clinical sign of loiasis [34].

Notion of a worm migrating through the eye is another pathognomonic symptom for loiasis, which was shown to occur more frequently in local Africans [32] where more than 40% of the population had the history of the worm migration [35]. Adult worms, 3–7 cm long, are visible in the subconjunctival space and cause the visual disturbances when they moved around the eye. Even though the worms under subconjunctiva could be removed under local anaesthetic with forceps, it is hard to extract them from the deep subconjunctival tissue [36]. In this study, two patients complained the discomfort of eyes with noticeable sensation of foreign objects, however, there was no conjunctival haemorrhage and diminution of vision observed. The worms did not reappear in the eye throughout hospitalization because of the fast migration of worm to the retrobulbar space. Routine ophthalmologic and funduscopic exam was often negative for eye worms.

In addition to eyes, adult worms (filaria) commonly reside in subcutaneous and deep connective tissue, but there is very limited literature describing the surgical removal of the intact *L. loa* adult worm and relevant pathological changes. In our study, two patients appeared with subcutaneous nodules under arm and chest. One of the nodules was removed in a skin biopsy procedure. A nematode worm section was clearly observed under microscope during the histochemical examination of the skin module. After being treated with ABZ + DEC, the size of the skin module in another patient reduced and disappeared. At present, two main strategies are utilized to manage loiasis: the surgical extraction of adult worms and the use of systemic antiparasitic medications [37]. Surgical intervention is pursued when adult worms are observable, such as during their migration beneath the conjunctiva. This is followed by the administration of antiparasitic drugs to eradicate microfilariae and any residual adult worms [38]. Nonetheless, the surgical removal has a limited effect on reducing the total number of worms in the host since these adult worms represent only a minor portion of the entire worm population. Consequently, irrespective of whether adult worm extraction is successful, systemic antiparasitic therapy is imperative to achieve a full recovery [39].

Except for the mild or nonspecific symptoms for those people infected with *L. loa* [14], the common symptom for people returned from loiasis endemic regions is the allergic type skin itching, which is more commonly reported in travelers or short term visitors than the local residents [40]. Studies demonstrated that pruritus occurred in 43.5% imported loiasis in immigrants in Spain from sub-Saharan Africa [15] and the symptom is persistent and all over the body. Pruritus is also common in this study and more than half of patients complained the skin itching that irritated and affected their sleep. Considering the higher incidence of loiasis in a population in endemic Gabon, the presence of “pruritus” combined with “frequent forest exposure” led to a large and nearly conclusive of disease-likelihood of loiasis [32].

*Loa loa* infection was associated with the presence of eosinophilia which occurs frequently in individuals returning from the endemic regions. Except for the positive predictive value for parasitic disease, eosinophils also play a crucial part in the fight against invasive helminthic infections. Among 154 returned travelers and migrants who had a total eosinophil count ≥ 500 cells/mL, 71 patients (46%) were diagnosed with helminthic infections [41, 42]. Although loiasis patients present mild or nonspecific symptoms, a significant number of them may reveal eosinophilia [14]. In our study, all of the *L. loa* infected individuals were significantly associated with absolute blood eosinophilia (0.54–40.30 × 10^9^/L). Recent findings demonstrate that eosinophils hinder parasite burden by initiating the rapid deployment of type 2 immune responses and producing major basic protein (MBP) to kill nematodes in the response to IL-5 [43]. An elevated presence of eosinophils and inflammatory responses were observed in a baboon model of hypermicrofilaremia [44]. Although immunoglobulins and eosinophils or others immune cells may be involved in killing microfilariae, the effector mechanism of eosinophils in controlling loiasis has barely been studied [45].

Apart from the presence of eosinophilia as a marker for helminth infections, microfilaraemia have been commonly observed as evidence of infection in residents of endemic regions [46]. The number of individuals with *L. loa* microfilaraemia may be expanded due to population growth in endemic areas [2]. The high load and persistent presence of *L. loa* microfilariae in the blood circulation could induce chronic pathogenic mechanisms which include obstructive or inflammatory processes in the vessels, or pathogenic processes induced by indirect immunologically mediated phenomena in various organs [47]. Consequently, severe disease outcomes were frequently occurred in individuals with high load of microfilariae [48]. Reports in the Republic of Congo suggest that chronic inflammation caused by eosinophilia may be related to excessive mortality [49]. Of note, the eosinophilia was more noticeable in loiasis with high load of microfilariae. A cross-sectional survey from Gabon showed that *L. loa* infection was associated with presence of eosinophilia and extent of microfilaraemia [50]. A patient from an endemic area, diagnosed with endomyocardial fibrosis, had *L. loa* microfilaraemia and marked eosinophilia [51]. However, it is still unknown whether *L. loa* microfilaraemia or eosinophilia is associated with any physiopathological change related to the infection.

Interestingly, some microfilaremic individuals from local residents were reported to get no sign of loiasis or *L. loa* infection. Possibly it is because these local people acquire immunity, especially Th-2 driven immune response that control the infection at low level and led to a lack of “reactive” symptoms in these individuals. Contrarily, travelers display a wide range of clinical manifestations when they visited the endemic areas and get infected because they lack the acquired immunity against the infection. The difference is more likely due to the distinct immunological profile [52, 53]. In addition, other studies claimed the presence of microfilaraemia may be related to the genetics of host and parasite and the density and fecundity of adult worms [54]. In this study, we found that all positive infective cases were associated with high absolute eosinophil counts and apparent disease manifestation, but only some of them displayed microfilaraemia, which was also observed in recent reports from Japan and China [23].

Although the laboratory identification of microfilaraemia and eosinophilia is important for the diagnosis of *L. loa* infection, a significant proportion of patients may suffer from occult loiasis with an absence of microfilaraemia and cross detection of antibodies limits the usefulness of serological test [32]. PCR assay plays a decisive important role in the diagnosis of imported loiasis, especially for those with low microfilariae load [55]. In a cross-sectional survey administered in Gabon, the higher detection rate of filarial infections was observed when PCR was applied in contrast to microscopy (48% vs. 20%, respectively) [56]. In our study, all patients were PCR positive regardless of whether they had microfilaraemia examined under microscope. As patients returned from endemic areas without microfilaraemia should be further tested with PCR to exclude *L. loa* or other filarial infections [55].

## Conclusions

As international travel and economic activities are increased, more imported loiasis cases are found in non-endemic countries. Based on the investigation on 21 cases with definitely diagnosed loiasis in Beijing, all of them had travel history to countries in sub-Saharan Africa, endemic regions for loiasis, during the past 6 months. The major clinical manifestations include Calabar swelling and predominant eosinophilia, sometimes accompanied by a variety of non-specific symptoms such as recurrent pruritus, muscle pain and skin rash. All cases showed positive in PCR detection in blood samples even though most infected people are lack of microfilaraemia. Phylogenetic analysis of the ITS1 sequences obtained from imported patients shows a closer relationship to those derived from *L. Loa*, and a more distant relation to other filariae such as *Onchocerca volvulus*, *Brugia malayi*, or *Dirofilaria repens*. This study provides useful information and guideline for physicians and researchers in non-endemic countries to diagnose and treat loiasis and *L. loa* infections acquired from endemic regions.

### Electronic supplementary material

Below is the link to the electronic supplementary material.

Supplementary Material 1

Supplementary Material 2

Supplementary Material 3

## Data Availability

All materials and data supporting these findings are involved within the manuscript and supplementary information files.
