# Supplementary figures and images for: Clinical and epidemiological features of imported loiasis in Beijing: a report from patients returned from Africa
Source: BMC Infect Dis. 2024 Jul 20;24:714. doi: 10.1186/s12879-024-09620-6 (PMC11265026; doi:10.1186/s12879-024-09620-6)

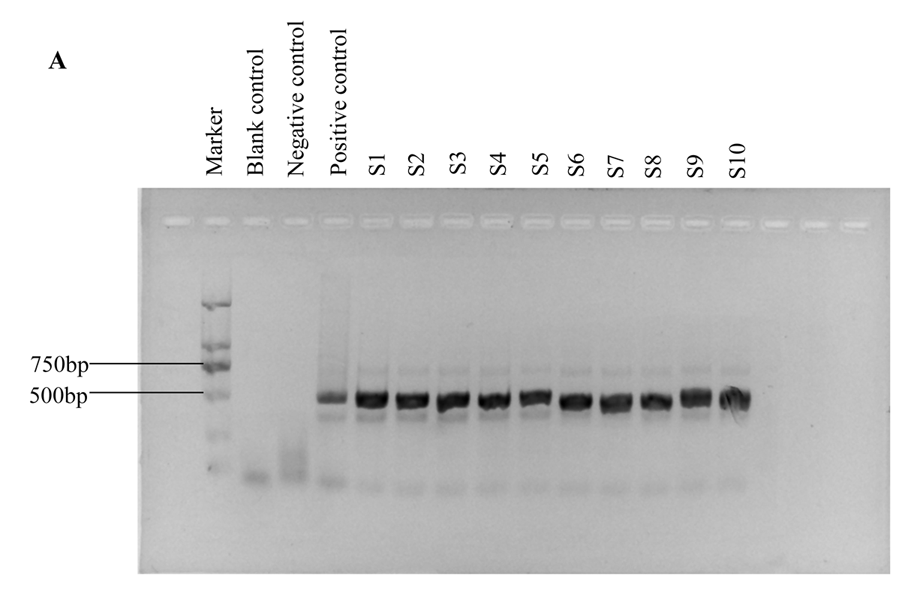

Supplement: Supplementary file 1 — Supplementary Material 1 [file 12879_2024_9620_MOESM1_ESM.tif]

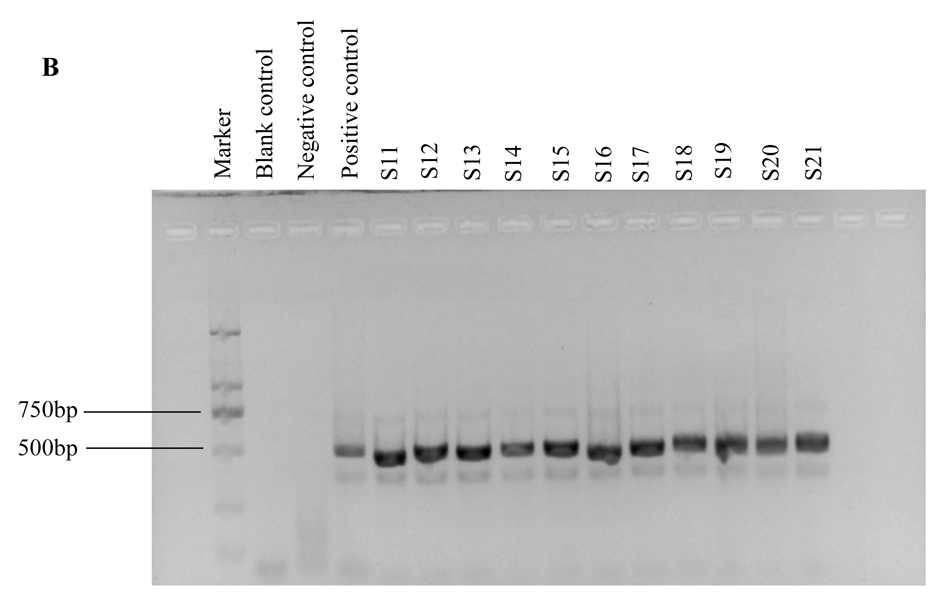

Supplement: Supplementary file 2 — Supplementary Material 2 [file 12879_2024_9620_MOESM2_ESM.tif]
