# Supplementary material for: Clinical and epidemiological features of imported loiasis in Beijing: a report from patients returned from Africa
Source: BMC Infect Dis. 2024 Jul 20;24:714. doi: 10.1186/s12879-024-09620-6 (PMC11265026; doi:10.1186/s12879-024-09620-6)
Supplement: Supplementary file 3 — Supplementary Material 3 [file 12879_2024_9620_MOESM3_ESM.docx]

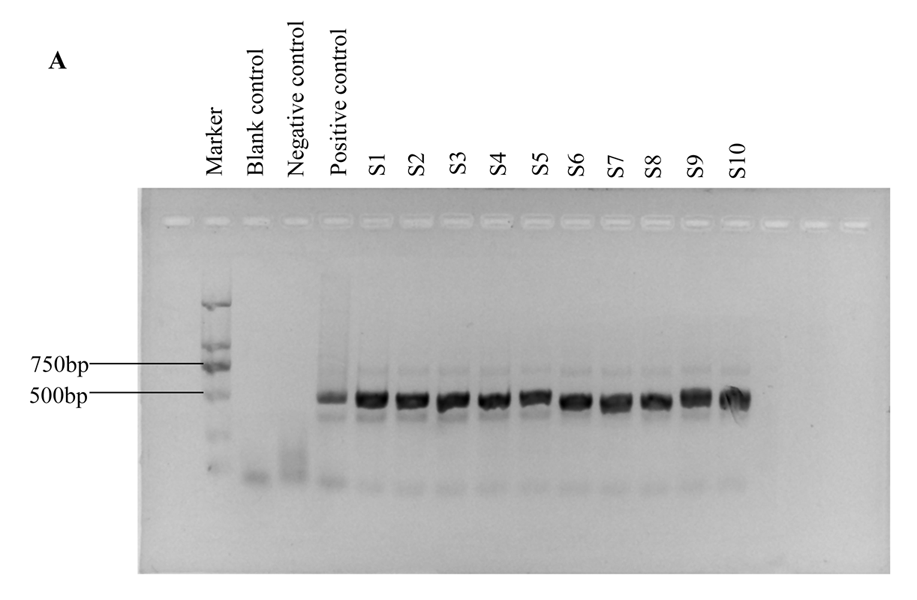


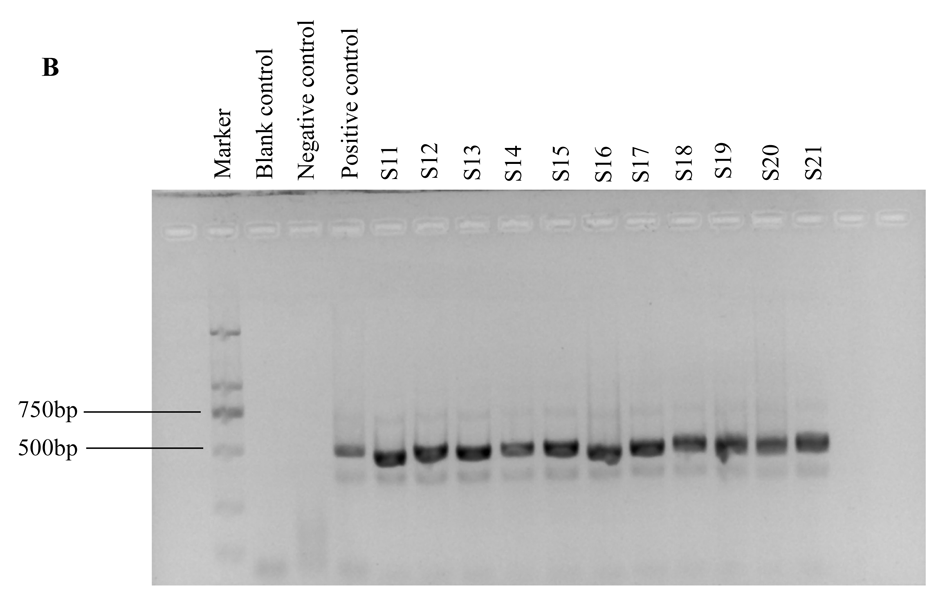


Additional file 1: Fig. S1 PCR products from the ITS1 region of blood samples from imported *L. loa* cases were amplified and identified. A total of 21 samples were assigned numbers S1 to S21. The gel electrophoresis results showed that 10 samples (S1-S10) were exhibited in gel image A, while the remaining 11 samples (S11-S21) were in gel image B. The marker on the left side of each gel represents the size of molecules, with L. loa having a fragment size of 457 base pairs. Gel image A and B also had controls including: Blank control (no DNA template), Negative control (distilled water as template) and Positive control (DNA of *L. loa* as template).
